# Synthesis and crystal structure of (1,4,7,10-tetra­aza­cyclo­dodecane-κ^4^ *N*)(tetra­sulfido-κ^2^ *S* ^1^,*S* ^4^)manganese(II)

**DOI:** 10.1107/S2056989020002492

**Published:** 2020-02-28

**Authors:** Felix Danker, Christian Näther, Wolfgang Bensch

**Affiliations:** a Institut für Anorganische Chemie, Universität Kiel, Max-Eyth. Str. 2, 241128 Kiel, Germany

**Keywords:** crystal structure, polysulfide, manganese(II), discrete complex, hydrogen bonding

## Abstract

The title compound, [Mn(S_4_)(C_8_H_20_N_4_)], was accidentally obtained by the hydro­thermal reaction of Mn(ClO_4_)_2_·6H_2_O, cyclen (cyclen = 1,4,7,10-tetra­aza­cyclo­dodeca­ne) and Na_3_SbS_4_·9H_2_O in water at 413 K, indicating that polysulfide anions might represent inter­mediates in the synthesis of thio­metallate compounds using Na_3_SbS_4_·9H_2_O as a reactant. X-ray powder diffraction proves that the sample is slightly contaminated with NaSb(OH)_6_ and an unknown crystalline phase. The crystal investigated was twinned with a twofold rotation axis as the twin element, and therefore a twin refinement using data in HKLF-5 format was performed. The asymmetric unit of the title compound consists of one Mn^II^ cation, one [S_4_]^2−^ anion and one cyclen ligand in general positions. The Mn^II^ cation is sixfold coordinated by two *cis*-S atoms of the [S_4_]^2−^ anions, as well as four N atoms of the cyclen ligand within an irregular coordination. The complexes are linked *via* pairs of N—H⋯S hydrogen bonds into chains, which are further linked into layers by additional N—H⋯S hydrogen bonding. These layers are connected into a three-dimensional network by inter­molecular N—H⋯S and C—H⋯S hydrogen bonding. It is noted that only one similar complex with Mn^II^ is reported in the literature.

## Abstract 2

In the crystal structure of the title compound, the Mn^II^ cations are sixfold coordinated by four N atoms of a cyclen ligand and two terminal S atoms of an [S_4_]^2−^ anion into discrete complexes that are connected *via* inter­molecular N—H⋯S hydrogen bonding into a three-dimensional network.

## Chemical context   

Investigations on the synthesis and crystal structures of new inorganic–organic chalcogenidometallates are an important topic in inorganic chemistry and many such compounds have been reported in the literature (Sheldrick & Wachhold, 1988 ▸; Dehnen & Melullis, 2007 ▸; Seidlhofer *et al.*, 2010 ▸, 2011 ▸; Wang *et al.*, 2016 ▸; Zhou, 2016 ▸; Zhu & Dai, 2017 ▸; Nie *et al.*, 2017 ▸). In this context, thio­anti­monates are of special inter­est because they show a variety of coordination numbers and can form networks of different dimensionality (Schur *et al.*, 2001 ▸; Jia *et al.*, 2004 ▸; Powell *et al.*, 2005 ▸; Zhang *et al.*, 2007 ▸; Liu & Zhou, 2011 ▸; Engelke *et al.*, 2004 ▸; Puls *et al.*, 2006 ▸). This is the reason why we have been inter­ested in this class of compounds for several years (Bensch *et al.*, 1997 ▸; Spetzler *et al.*, 2004 ▸, 2005 ▸; Stähler *et al.*, 2001 ▸; Lühmann *et al.*, 2008 ▸). Most of these compounds were synthesized by solvothermal reactions using the elements as reactands, which is a disadvantage for several reasons. Recently, we have found that many such compounds are more easily available if simple metal salts such as, for example, Schlippe’s salt (Na_3_SbS_4_·9H_2_O) or NaSbS_3_ are used as starting materials (Anderer *et al.*, 2014 ▸, 2016 ▸; Danker *et al.*, 2020 ▸). The major advantage of this approach is the fact that different SbS_*x*_ species are present in solution, which in some cases allows the preparation of thio­anti­monates already at room temperature. The reactions in solution are complex, but it has been found that Schlippe’s salt is unstable and forms different species such as, for example, [SbS_3_O]^3−^, HS^−^, [S_2_O_3_]^2−^ or [SbS_4_]^3−^ anions (Anderer *et al.*, 2014 ▸; Long *et al.*, 1970 ▸; Rammelsberg, 1841 ▸; Planer-Friedrich & Wilson, 2012 ▸; Planer-Friedrich & Scheinost, 2011 ▸; Mosselmans *et al.*, 2000 ▸).
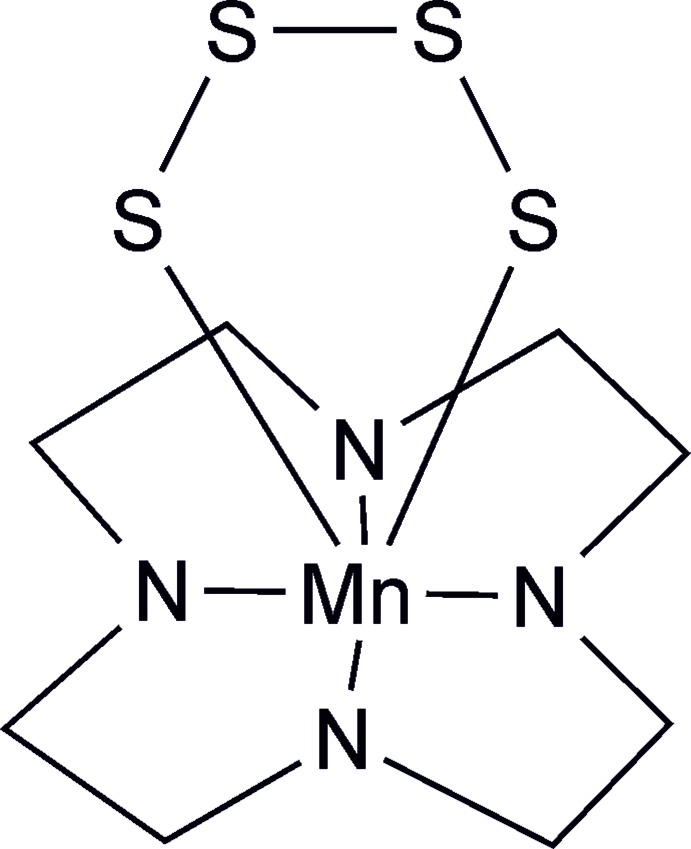


In the course of our investigations we became inter­ested in compounds based on cyclen as the ligand (cyclen = 1,4,7,10-tetra­aza­cyclo­dodeca­ne); cyclen is a tetra­dentate ligand that in an octa­hedral coordination provides two free coordination sites that can be used by the metal cation to connect to a thio­anti­monate network. In this context, Mn^II^ cations are of special inter­est because this cation exhibits a high affinity to sulfur. Therefore, Na_3_SbS_4_·9H_2_O was reacted with manganese perchlorate under hydro­thermal conditions leading to yellow plate-like crystals, which were identified by single crystal X-ray diffraction. Surprisingly, the structure consists of discrete complexes, in which manganese is coordinated by one cyclen ligand and one tetra­sulfide dianion that must have formed *in situ* from Na_3_SbS_4_. This finding is of special inter­est because it indicates that polysulfide species might represent inter­mediates in the synthesis of thio­metallate compounds using Na_3_SbS_4_ as reactant. It is noted that only one similar complex has been reported in the literature, in which the Mn^II^ cations are linked to a tridentate chelating ligand, one water mol­ecule and one tetra­sulfide dianion, which was synthesized by a completely different route (Wieghardt *et al.*, 1987 ▸).

**Figure 1:**
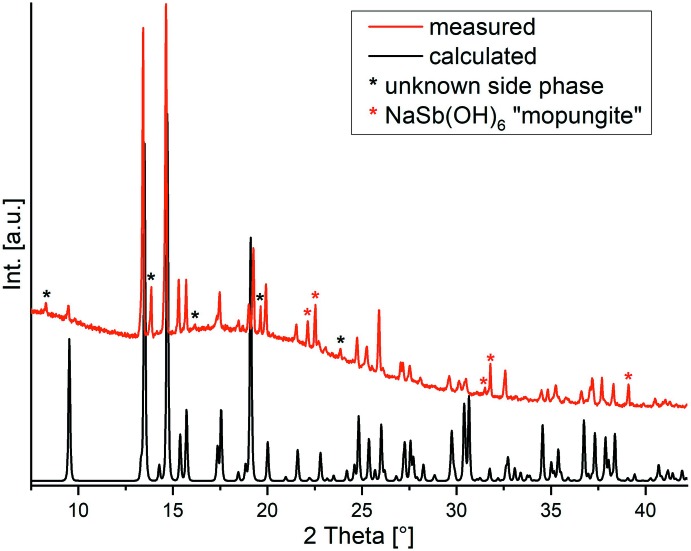
Experimental and calculated XRPD powder patterns of the title compound. The reflections of side products are marked by stars.

Investigations using X-ray powder diffraction (XRPD) proved that the title compound was obtained as the major phase but is contaminated with small amount of mopungite [NaSb(OH)_6_; Schrewelius, 1938 ▸; Asai, 1975 ▸) and an additional crystalline phase of unknown identity (Fig. 1 ▸). The title compound cannot be obtained as a pure crystalline phase if the reaction conditions are varied and therefore, no further investigations were performed.

## Structural commentary   

**Figure 2:**
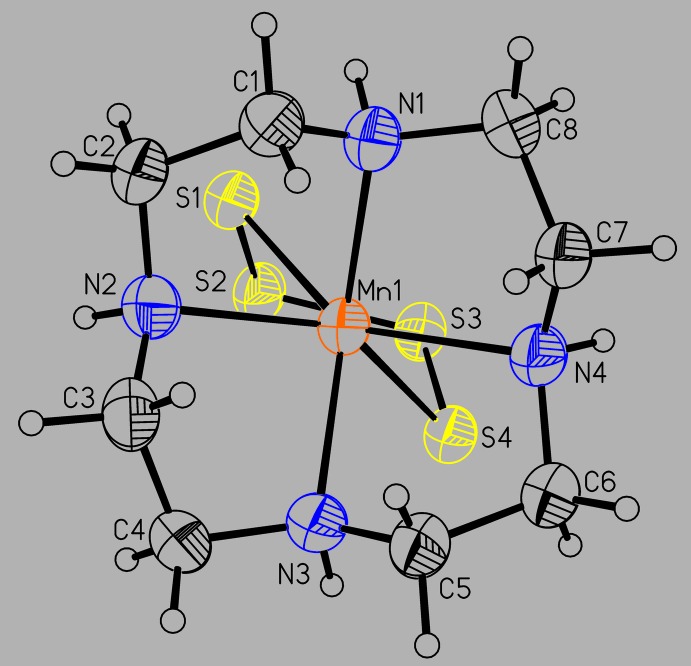
Molecular structure of the title compound with atom labelling and displacement ellipsoids drawn at the 50% probability level.

**Figure 3:**
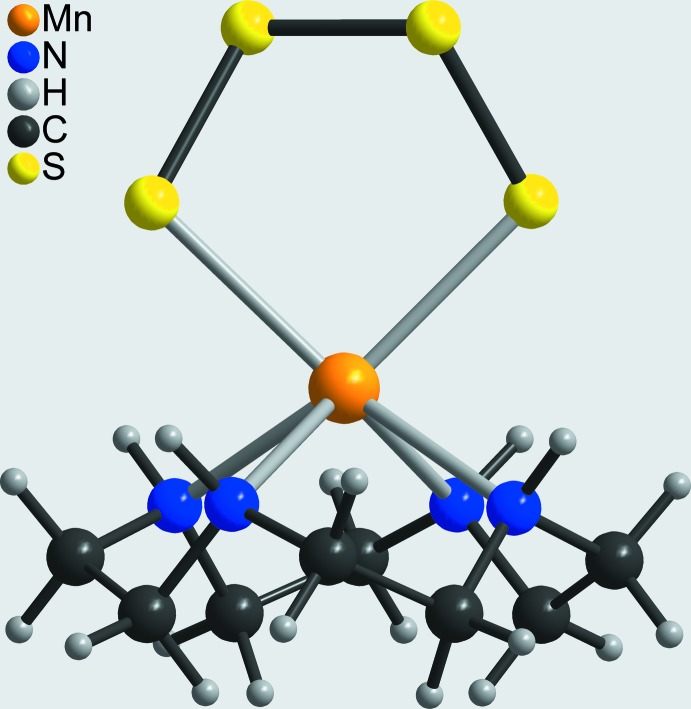
View of the Mn coordination sphere in the molecular structure of the title compound.

The asymmetric unit of the title compound consists of one Mn^II^ cation, one tetra­sulfido anion and one cyclen ligand in general positions. The Mn^II^ cations are coordinated by two terminal S atoms of the tetra­sulfido anion and the N atoms of the cyclen ligand (Fig. 2 ▸). The Mn—N bond lengths range from 2.294 (5) to 2.329 (4) Å, which corresponds to literature values (Table 1 ▸). The Mn—S bond lengths of 2.5894 (2) and 2.6195 (2) Å (Table 1 ▸) are slightly longer that those in the similar complex aqua-(μ-1,4-tetra­sulfido)*N*,*N*′,*N*′′-trimethyl-1,4,7-tri­aza­cyclo­nona­nemanganese(II) (Wieghardt *et al.*, 1987 ▸). The [S_4_]^2−^ anion shows a staggered conformation with a value of the torsion angle along the S atoms of 61.7 (6)°. The bond angles within this complex are far from the ideal values, which shows that the Mn^II^ cations are in an irregular coordination (Fig. 3 ▸ and Table 1 ▸). This arises for steric reasons, because the Mn^II^ cation is located 1.149 (1) Å above the plane formed by the cyclene N atoms and the terminal S atoms of the tetra­sulfido anion are enforced to be in *cis*-positions.

## Supra­molecular features   

**Figure 4:**
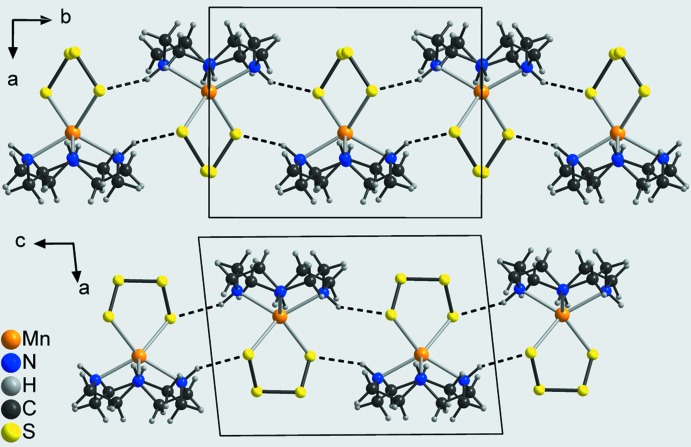
View of the chains running along the *b*- (top) and the *c*-axis (bottom) directions with inter­molecular N—H⋯S hydrogen bonds shown as dashed lines.

**Figure 5:**
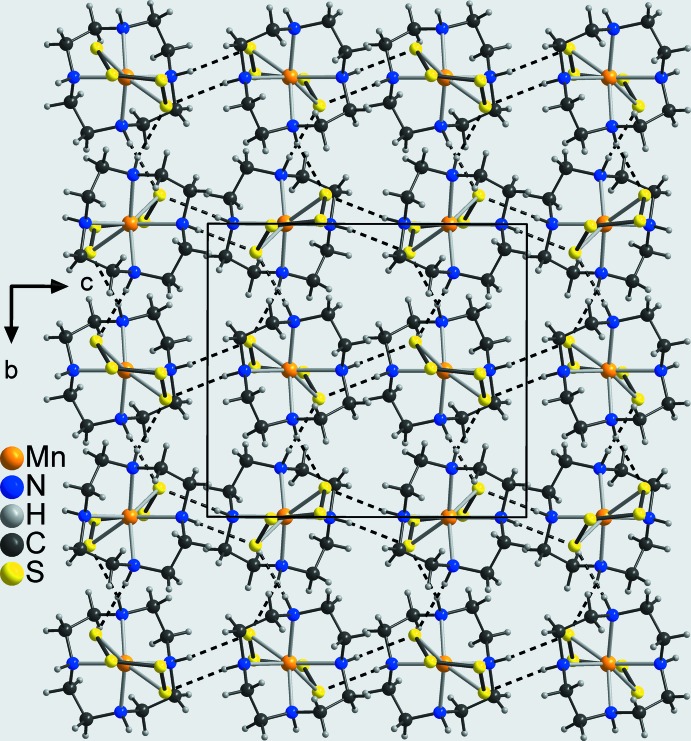
Crystal structure of the title compound with view of the layers along the *a*-axis direction with inter­molecular N—H⋯S hydrogen bonds shown as dashed lines.

**Figure 6:**
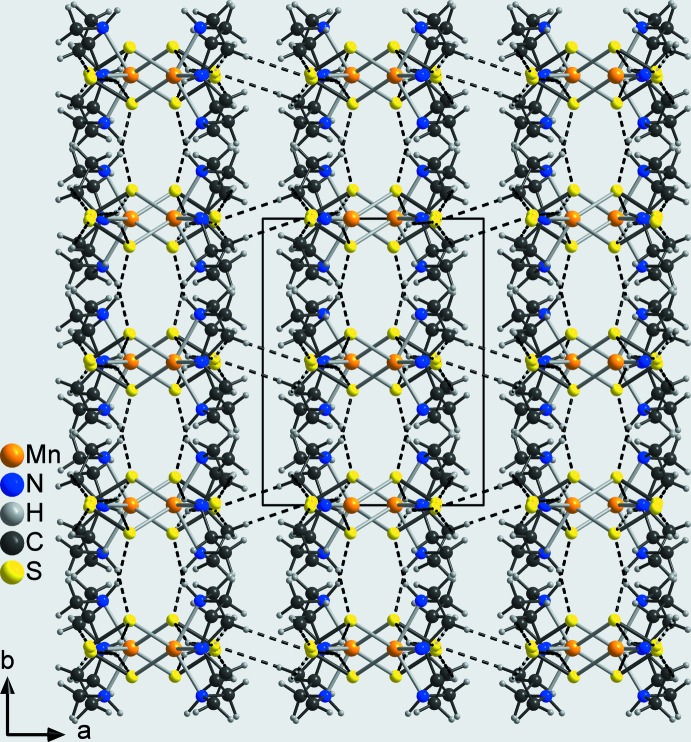
Crystal structure of the title compound viewed in the direction of the layers along the *c* axis with inter­molecular N—H⋯S and C—H⋯S hydrogen bonds shown as dashed lines.

In the crystal structure of the title compound, the discrete complexes are linked by pairs of N—H⋯S hydrogen bonds between atom S4 (H3) of one complex and H1 (S1) of a neighbouring complex into eight-membered rings that are condensed into chains propagating in the *b*-axis direction (Fig. 4 ▸: top and Table 2 ▸). The H⋯S distances of 2.50 and 2.48 Å and the N—H⋯S angles of 148 and 152° indicate a relatively strong inter­action (Table 2 ▸). The terminal S atoms S4 of two neighbouring complexes act as acceptors for a second hydrogen bond to the amino H atoms H4 of these complexes, also forming eight-membered rings that in this case are located on centers of inversion (Fig. 4 ▸: bottom and Table 2 ▸). These rings are condensed into chains that propagate along the *c*-axis direction (Fig. 4 ▸: bottom). As each complex is part of both of these two chains, layers are formed parallel to the *bc* plane (Fig. 5 ▸). The layers are linked into a three-dimensional network by C—H⋯S and additional N—H⋯S hydrogen bonding (Fig. 6 ▸ and Table 2 ▸).

## Database survey   

There is only one crystal structure reported in which Mn^II^ cations are linked to [S_4_]^2−^ anions and this compound was obtained from the reaction of manganese acetate with ammonium sulfide. This structure is similar to that of the title compound, but in this case the Mn^II^ cation is linked to a tridentate N-donor ligand and the Mn coordination is completed by one water mol­ecule (Wieghardt *et al.*, 1987 ▸). Complexes with other transition-metal cations that are related to the structure of the title compound are not reported in the Cambridge Structural Database (Version 2020; Groom *et al.*, 2016 ▸). For Zn and Ni, one complex is found in which the Ni cations are in a square-pyramidal coordination of four S atoms of two [S_4_]^2−^ anions and charge balance is achieved by tetra­ethyl­ammonium cations (Müller *et al.*, 1983 ▸; (Coucouvanis *et al.*, 1985 ▸). Similar compounds are also reported with Ni and Hg, but the tetra­ethyl­ammonium cations are replaced by tetra­phenyl­phospho­nium cations (Coucouvanis *et al.*, 1985 ▸; Müller *et al.*, 1985 ▸; Bailey *et al.*, 1991 ▸).

## Synthesis and crystallization   

**Synthesis of Na_3_SbS_4_·9H_2_O:**

Na_3_SbS_4_·9H_2_O was synthesized by adding 16.6 g (0.213 mol) of Na_2_S·*x*H_2_O (technical grade, purchased from Acros Organics) to 58 mL of demineralized water. This solution was heated to 323 K for 1h. Afterwards 19.6 g (0.058 mol) of Sb_2_S_3_ (98%, purchased from Alfa Aesar) and 3.69 g (0.115 mol) of sulfur (min. 99%, purchased from Alfa Aesar), were added and the reaction mixture was heated to 343 K for 6 h. The reaction mixture was filtered and the filtrate was stored overnight, leading to the formation of slightly yellow crystals, that were filtered off, washed with small amounts of water and dried under vacuum (yield about 30% based on Sb_2_S_3_).

**Synthesis of the title compound:**

The title compound was synthesized by the reaction of 36.8 mg (0.1 mmol) of Mn(ClO_4_)_2_·6H_2_O (99%, purchased from Alfa Aesar), 17.2 mg (0.1 mmol) of cyclen (98%, purchased from Strem Chemicals) and 288.8 mg (0.6 mmol) of Na_3_SbS_4_·9H_2_O. The reaction mixture was heated at 413 K for 11 d in 2 mL of water, leading to the formation of a precipitate that was filtered off. XRPD investigations proved the product to consist of the title compound as the major phase and very small amounts of NaSb(OH)_6_ and an additional crystalline phase of unknown identity.

**Experimental methods:**

The XRPD measurements were performed using a Stoe Transmission Powder Diffraction System (STADI P) with Cu *K*a radiation that was equipped with a linear, position-sensitive MYTHEN detector from Stoe & Cie.

## Refinement   

Crystal data, data collection and structure refinement details are summarized in Table 3 ▸. Hydrogen atoms were positioned with idealized geometry (N—H = 1.00 Å, C—H = 0.99 Å) and were refined using a riding model with *U*_iso_(H) = 1.2*U*_eq_(C). In the first refinements, poor reliability factors and several residual electron densities were found in the difference map, for which no reasonable structure model can be found, indicating twinning. *PLATON* (Spek, 2020 ▸) immediately detected a pseudo-twofold rotation axis as the twin element, indicating non-merohedral twinning. Therefore, the data were transformed into HKLF-5 format and a twin refinement was performed, leading to a BASF parameter of 0.473 (5) and a significant improvement of all reliability factors. *PLATON* detected pseudo symmetry but investigations showed the unit cell and space group to be correct. Please note that symmetry-equivalent reflections had to be be merged before refinement and thus no *R*_int_ value can be given.

## Supplementary Material

Crystal structure: contains datablock(s) I. DOI: 10.1107/S2056989020002492/lh5948sup1.cif

Structure factors: contains datablock(s) I. DOI: 10.1107/S2056989020002492/lh5948Isup2.hkl

CCDC reference: 1985538

Additional supporting information:  crystallographic information; 3D view; checkCIF report

## Figures and Tables

**Table 1:** Selected geometric parameters (Å, °)

Mn1—N1	2.294 (5)	Mn1—N4	2.329 (4)
Mn1—N3	2.313 (5)	Mn1—S4	2.5894 (17)
Mn1—N2	2.317 (5)	Mn1—S1	2.6195 (16)
			
N1—Mn1—N3	120.76 (17)	N2—Mn1—S4	145.61 (13)
N1—Mn1—N2	76.68 (16)	N4—Mn1—S4	83.16 (13)
N3—Mn1—N2	74.77 (16)	N1—Mn1—S1	86.82 (12)
N1—Mn1—N4	74.82 (15)	N3—Mn1—S1	137.52 (11)
N3—Mn1—N4	76.56 (16)	N2—Mn1—S1	82.28 (12)
N2—Mn1—N4	120.07 (17)	N4—Mn1—S1	145.49 (13)
N1—Mn1—S4	136.95 (12)	S4—Mn1—S1	91.36 (5)
N3—Mn1—S4	88.18 (13)		

**Table 2:** Hydrogen-bond geometry (Å, °)

*D*—H⋯*A*	*D*—H	H⋯*A*	*D*⋯*A*	*D*—H⋯*A*
N1—H1⋯S4^i^	1.00	2.50	3.389 (5)	148
N2—H2⋯S1^ii^	1.00	2.63	3.514 (4)	147
C3—H3*A*⋯S2^ii^	0.99	2.98	3.744 (5)	135
N3—H3⋯S1^iii^	1.00	2.48	3.394 (5)	152
N4—H4⋯S3^iv^	1.00	2.97	3.534 (4)	117
N4—H4⋯S4^iv^	1.00	2.64	3.570 (5)	154
C7—H7*A*⋯S3^iv^	0.99	2.98	3.699 (5)	130
C7—H7*B*⋯S3^v^	0.99	2.98	3.756 (6)	136

Symmetry codes: (i) 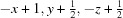; (ii) 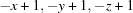; (iii) 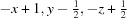; (iv) 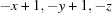; (v) 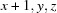.

**Table 3:** Experimental details

Crystal data	
Chemical formula	[Mn(S_4_)(C_8_H_20_N_4_)]
*M*_r_	355.46
Crystal system, space group	Monoclinic, *P*2_1_/*c*
Temperature (K)	170
*a*, *b*, *c* (Å)	9.3292 (6), 12.0371 (5), 13.1750 (8)
β (°)	95.885 (5)
*V* (Å^3^)	1471.71 (14)
*Z*	4
Radiation type	Mo *K*α
μ (mm^−1^)	1.45
Crystal size (mm)	0.15 × 0.15 × 0.05

Data collection	
Diffractometer	Stoe IPDS2
Absorption correction	Numerical (*X-RED32* and *X-SHAPE*; Stoe & Cie, 2008 ▸)
*T*_min_, *T*_max_	0.704, 0.873
No. of measured, independent and observed [*I* > 2σ(*I*)] reflections	2959, 2959, 1919
(sin θ/λ)_max_ (Å^−1^)	0.624

Refinement	
*R*[*F* ^2^ > 2σ(*F* ^2^)], *wR*(*F* ^2^), *S*	0.063, 0.173, 1.03
No. of reflections	2959
No. of parameters	155
H-atom treatment	H-atom parameters constrained
Δρ_max_, Δρ_min_ (e Å^−3^)	0.57, −0.70

Computer programs: *X-AREA* (Stoe & Cie, 2008 ▸), *SHELXS97* (Sheldrick, 2008 ▸), *SHELXL2018/3* (Sheldrick, 2015 ▸), *DIAMOND* (Brandenburg, 1999 ▸) and *publCIF* (Westrip, 2010 ▸).

## supplementary crystallographic information

### Crystal data

**Table:**

[Mn(S_4_)(C_8_H_20_N_4_)]	*F*(000) = 740
*M**_r_* = 355.46	*D*_x_ = 1.604 Mg m^−^^3^
Monoclinic, *P*2_1_/*c*	Mo *K*α radiation, λ = 0.71073 Å
*a* = 9.3292 (6) Å	Cell parameters from 9562 reflections
*b* = 12.0371 (5) Å	θ = 2.2–26.3°
*c* = 13.1750 (8) Å	µ = 1.45 mm^−^^1^
β = 95.885 (5)°	*T* = 170 K
*V* = 1471.71 (14) Å^3^	Plate, yellow
*Z* = 4	0.15 × 0.15 × 0.05 mm

### Data collection

**Table:**

STOE IPDS-2 diffractometer	1919 reflections with *I* > 2σ(*I*)
ω scans	θ_max_ = 26.3°, θ_min_ = 2.2°
Absorption correction: numerical (X-Red32 and X-Shape; Stoe & Cie, 2008)	*h* = −11→11
*T*_min_ = 0.704, *T*_max_ = 0.873	*k* = −14→14
2959 measured reflections	*l* = −14→16
2959 independent reflections	

### Refinement

**Table:**

Refinement on *F*^2^	0 restraints
Least-squares matrix: full	Hydrogen site location: inferred from neighbouring sites
*R*[*F*^2^ > 2σ(*F*^2^)] = 0.063	H-atom parameters constrained
*wR*(*F*^2^) = 0.173	*w* = 1/[σ^2^(*F*_o_^2^) + (0.0882*P*)^2^] where *P* = (*F*_o_^2^ + 2*F*_c_^2^)/3
*S* = 1.02	(Δ/σ)_max_ < 0.001
2959 reflections	Δρ_max_ = 0.57 e Å^−^^3^
155 parameters	Δρ_min_ = −0.70 e Å^−^^3^

### Special details

**Table:**

Geometry. All esds (except the esd in the dihedral angle between two l.s. planes) are estimated using the full covariance matrix. The cell esds are taken into account individually in the estimation of esds in distances, angles and torsion angles; correlations between esds in cell parameters are only used when they are defined by crystal symmetry. An approximate (isotropic) treatment of cell esds is used for estimating esds involving l.s. planes.
Refinement. Refined as a two-component twin

### Fractional atomic coordinates and isotropic or equivalent isotropic displacement parameters (Å^2^)

**Table:**

	*x*	*y*	*z*	*U*_iso_*/*U*_eq_	
Mn1	0.59848 (9)	0.49918 (7)	0.25694 (6)	0.0422 (3)	
N1	0.7171 (5)	0.6664 (4)	0.2664 (3)	0.0457 (11)	
H1	0.645621	0.725211	0.279659	0.055*	
C1	0.8299 (6)	0.6651 (5)	0.3543 (4)	0.0469 (13)	
H1A	0.861723	0.741829	0.371547	0.056*	
H1B	0.914309	0.621997	0.336767	0.056*	
C2	0.7676 (6)	0.6122 (5)	0.4446 (4)	0.0468 (13)	
H2A	0.842206	0.610102	0.503699	0.056*	
H2B	0.686271	0.657868	0.463784	0.056*	
N2	0.7167 (5)	0.4989 (4)	0.4204 (3)	0.0477 (11)	
H2	0.644962	0.478937	0.468826	0.057*	
C3	0.8319 (6)	0.4142 (5)	0.4303 (4)	0.0496 (14)	
H3A	0.861502	0.399186	0.503323	0.060*	
H3B	0.917018	0.441783	0.398780	0.060*	
C4	0.7769 (7)	0.3086 (5)	0.3777 (4)	0.0525 (14)	
H4A	0.855111	0.252813	0.380371	0.063*	
H4B	0.697143	0.277493	0.413007	0.063*	
N3	0.7251 (5)	0.3338 (4)	0.2703 (3)	0.0452 (11)	
H3	0.657863	0.273002	0.244550	0.054*	
C5	0.8434 (6)	0.3386 (5)	0.2033 (4)	0.0468 (13)	
H5A	0.880323	0.262817	0.192812	0.056*	
H5B	0.923624	0.384099	0.236072	0.056*	
C6	0.7886 (7)	0.3891 (5)	0.1014 (4)	0.0495 (14)	
H6A	0.868193	0.393285	0.057310	0.059*	
H6B	0.711996	0.341272	0.067012	0.059*	
N4	0.7302 (5)	0.5024 (4)	0.1160 (3)	0.0458 (11)	
H4	0.661531	0.519747	0.054772	0.055*	
C7	0.8394 (6)	0.5908 (5)	0.1258 (4)	0.0477 (14)	
H7A	0.871251	0.607700	0.058040	0.057*	
H7B	0.924346	0.566065	0.171399	0.057*	
C8	0.7750 (7)	0.6935 (5)	0.1692 (4)	0.0505 (14)	
H8A	0.849852	0.751719	0.180854	0.061*	
H8B	0.696785	0.722648	0.119904	0.061*	
S1	0.39710 (16)	0.59715 (13)	0.34907 (10)	0.0482 (4)	
S2	0.21580 (17)	0.50670 (14)	0.30042 (11)	0.0524 (4)	
S3	0.22169 (17)	0.48858 (14)	0.14646 (10)	0.0523 (4)	
S4	0.40779 (16)	0.40112 (13)	0.13102 (10)	0.0478 (4)	

### Atomic displacement parameters (Å^2^)

**Table:**

	*U*^11^	*U*^22^	*U*^33^	*U*^12^	*U*^13^	*U*^23^
Mn1	0.0532 (5)	0.0398 (5)	0.0347 (4)	0.0000 (4)	0.0106 (3)	0.0007 (3)
N1	0.053 (3)	0.047 (3)	0.039 (2)	0.000 (2)	0.0138 (19)	−0.0016 (19)
C1	0.055 (3)	0.044 (3)	0.043 (3)	−0.005 (3)	0.009 (2)	−0.003 (2)
C2	0.060 (3)	0.048 (3)	0.034 (2)	−0.001 (3)	0.011 (2)	−0.004 (2)
N2	0.059 (3)	0.045 (3)	0.041 (2)	0.001 (2)	0.016 (2)	0.002 (2)
C3	0.060 (4)	0.055 (4)	0.034 (2)	0.005 (3)	0.005 (2)	0.004 (2)
C4	0.073 (4)	0.042 (3)	0.044 (3)	0.009 (3)	0.012 (3)	0.008 (3)
N3	0.055 (3)	0.039 (3)	0.043 (2)	−0.002 (2)	0.010 (2)	−0.0001 (19)
C5	0.056 (3)	0.047 (3)	0.039 (3)	0.008 (3)	0.010 (2)	0.000 (2)
C6	0.063 (4)	0.047 (3)	0.041 (3)	0.005 (3)	0.016 (2)	−0.001 (2)
N4	0.056 (3)	0.043 (3)	0.040 (2)	−0.006 (2)	0.015 (2)	−0.0024 (19)
C7	0.064 (4)	0.044 (3)	0.038 (2)	−0.003 (3)	0.019 (2)	−0.001 (2)
C8	0.066 (4)	0.047 (3)	0.040 (3)	−0.002 (3)	0.011 (3)	0.009 (3)
S1	0.0562 (9)	0.0480 (9)	0.0418 (7)	0.0009 (7)	0.0114 (6)	−0.0023 (6)
S2	0.0593 (9)	0.0558 (10)	0.0443 (7)	−0.0032 (8)	0.0167 (6)	−0.0025 (6)
S3	0.0606 (9)	0.0551 (10)	0.0414 (7)	0.0035 (8)	0.0061 (6)	−0.0003 (6)
S4	0.0567 (9)	0.0473 (9)	0.0408 (7)	−0.0015 (7)	0.0118 (6)	−0.0022 (6)

### Geometric parameters (Å, º)

**Table:**

Mn1—N1	2.294 (5)	C4—H4A	0.9900
Mn1—N3	2.313 (5)	C4—H4B	0.9900
Mn1—N2	2.317 (5)	N3—C5	1.483 (6)
Mn1—N4	2.329 (4)	N3—H3	1.0000
Mn1—S4	2.5894 (17)	C5—C6	1.513 (8)
Mn1—S1	2.6195 (16)	C5—H5A	0.9900
N1—C8	1.477 (6)	C5—H5B	0.9900
N1—C1	1.482 (7)	C6—N4	1.488 (7)
N1—H1	1.0000	C6—H6A	0.9900
C1—C2	1.517 (7)	C6—H6B	0.9900
C1—H1A	0.9900	N4—C7	1.470 (7)
C1—H1B	0.9900	N4—H4	1.0000
C2—N2	1.468 (7)	C7—C8	1.513 (8)
C2—H2A	0.9900	C7—H7A	0.9900
C2—H2B	0.9900	C7—H7B	0.9900
N2—C3	1.477 (7)	C8—H8A	0.9900
N2—H2	1.0000	C8—H8B	0.9900
C3—C4	1.512 (8)	S1—S2	2.058 (2)
C3—H3A	0.9900	S2—S3	2.0465 (19)
C3—H3B	0.9900	S3—S4	2.058 (2)
C4—N3	1.480 (7)		
			
N1—Mn1—N3	120.76 (17)	N3—C4—H4A	109.8
N1—Mn1—N2	76.68 (16)	C3—C4—H4A	109.8
N3—Mn1—N2	74.77 (16)	N3—C4—H4B	109.8
N1—Mn1—N4	74.82 (15)	C3—C4—H4B	109.8
N3—Mn1—N4	76.56 (16)	H4A—C4—H4B	108.3
N2—Mn1—N4	120.07 (17)	C4—N3—C5	112.8 (5)
N1—Mn1—S4	136.95 (12)	C4—N3—Mn1	111.3 (3)
N3—Mn1—S4	88.18 (13)	C5—N3—Mn1	109.0 (3)
N2—Mn1—S4	145.61 (13)	C4—N3—H3	107.8
N4—Mn1—S4	83.16 (13)	C5—N3—H3	107.8
N1—Mn1—S1	86.82 (12)	Mn1—N3—H3	107.8
N3—Mn1—S1	137.52 (11)	N3—C5—C6	109.8 (5)
N2—Mn1—S1	82.28 (12)	N3—C5—H5A	109.7
N4—Mn1—S1	145.49 (13)	C6—C5—H5A	109.7
S4—Mn1—S1	91.36 (5)	N3—C5—H5B	109.7
C8—N1—C1	112.7 (4)	C6—C5—H5B	109.7
C8—N1—Mn1	111.3 (3)	H5A—C5—H5B	108.2
C1—N1—Mn1	109.4 (3)	N4—C6—C5	110.3 (4)
C8—N1—H1	107.7	N4—C6—H6A	109.6
C1—N1—H1	107.7	C5—C6—H6A	109.6
Mn1—N1—H1	107.7	N4—C6—H6B	109.6
N1—C1—C2	108.6 (4)	C5—C6—H6B	109.6
N1—C1—H1A	110.0	H6A—C6—H6B	108.1
C2—C1—H1A	110.0	C7—N4—C6	114.5 (4)
N1—C1—H1B	110.0	C7—N4—Mn1	111.1 (3)
C2—C1—H1B	110.0	C6—N4—Mn1	108.5 (3)
H1A—C1—H1B	108.4	C7—N4—H4	107.4
N2—C2—C1	111.1 (4)	C6—N4—H4	107.4
N2—C2—H2A	109.4	Mn1—N4—H4	107.4
C1—C2—H2A	109.4	N4—C7—C8	109.0 (4)
N2—C2—H2B	109.4	N4—C7—H7A	109.9
C1—C2—H2B	109.4	C8—C7—H7A	109.9
H2A—C2—H2B	108.0	N4—C7—H7B	109.9
C2—N2—C3	114.0 (5)	C8—C7—H7B	109.9
C2—N2—Mn1	108.2 (3)	H7A—C7—H7B	108.3
C3—N2—Mn1	110.8 (3)	N1—C8—C7	110.0 (5)
C2—N2—H2	107.9	N1—C8—H8A	109.7
C3—N2—H2	107.9	C7—C8—H8A	109.7
Mn1—N2—H2	107.9	N1—C8—H8B	109.7
N2—C3—C4	109.2 (5)	C7—C8—H8B	109.7
N2—C3—H3A	109.8	H8A—C8—H8B	108.2
C4—C3—H3A	109.8	S2—S1—Mn1	102.89 (7)
N2—C3—H3B	109.8	S3—S2—S1	105.09 (9)
C4—C3—H3B	109.8	S2—S3—S4	105.14 (9)
H3A—C3—H3B	108.3	S3—S4—Mn1	103.57 (7)
N3—C4—C3	109.2 (4)		

### Hydrogen-bond geometry (Å, º)

**Table:**

*D*—H···*A*	*D*—H	H···*A*	*D*···*A*	*D*—H···*A*
N1—H1···S4^i^	1.00	2.50	3.389 (5)	148
N2—H2···S1^ii^	1.00	2.63	3.514 (4)	147
C3—H3*A*···S2^ii^	0.99	2.98	3.744 (5)	135
N3—H3···S1^iii^	1.00	2.48	3.394 (5)	152
N4—H4···S3^iv^	1.00	2.97	3.534 (4)	117
N4—H4···S4^iv^	1.00	2.64	3.570 (5)	154
C7—H7*A*···S3^iv^	0.99	2.98	3.699 (5)	130
C7—H7*B*···S3^v^	0.99	2.98	3.756 (6)	136

Symmetry codes: (i) −*x*+1, *y*+1/2, −*z*+1/2; (ii) −*x*+1, −*y*+1, −*z*+1; (iii) −*x*+1, *y*−1/2, −*z*+1/2; (iv) −*x*+1, −*y*+1, −*z*; (v) *x*+1, *y*, *z*.
